# Effect of 6% hydroxyethyl starch-450 and low molecular weight dextran on blood sugar levels during surgery under subarachnoid block: A prospective randomised study

**DOI:** 10.4103/0019-5049.71045

**Published:** 2010

**Authors:** Abhiruchi Patki, VC Shelgaonkar

**Affiliations:** Department of Anaesthesiology, Indira Gandhi Medical College and Mayo Hospital, Nagpur, India

**Keywords:** 6% hydroxyethyl starch-450, capillary blood glucose, dextran-40, hyperglycaemia, preloading, Ringer’s lactate, spinal anaesthesia

## Abstract

Dextrans and hydroxyethyl starches produce significant levels of free glucose residues following metabolism. The following study was designed to compare 6% hydroxyethyl starch-450 with Dextran 40, both used as preloading fluids, for their potential to raise peri-operative blood glucose levels. After taking an informed consent, 180 non-diabetic adult patients, posted for elective surgery under spinal anaesthesia, were randomly divided into three groups, to receive Ringer’s Lactate 20 ml/kg (group 1), Dextran 40,10 ml/kg (group 2) and Hestar 6%-450, 10 ml/kg (group 3), over half an hour, prior to the subarachnoid block, as preloading fluid, and serial capillary blood glucose measurements were taken thereafter at regular intervals up to 240 minutes from the baseline reading. All the three preloading fluids, including Ringer’s Lactate used as control, were seen to significantly increase the capillary blood glucose levels intra-operatively (*P* < 0.05), but the rise with Dextran-40 was seen to be sustained and highly significant (*P* < 0.001). We thus conclude that, Dextran40 causes a sustained and significant rise in peri-operative blood glucose levels.

## INTRODUCTION

Fluid resuscitation for hypovolaemic shock is an integral part of the acute medical management in an Intensive Care Unit (ICU) or inside an Operating Room (OR), and, commercially available synthetic colloids are widely used to serve this purpose, having been recommended in a number of resuscitation guidelines and intensive care algorithms.[1–4]

Colloids are also used as preloading fluids prophylactically, to limit complications following sympathetic blockade in central neuraxis blockade.[5–7] Colloid-crystalloid combinations are regularly being used as priming fluids in cardiopulmonary bypass circuits as well.[8,9]

Dextrans are polysaccharides that are normally broken down completely to carbon dioxide and water by the enzyme dextranase, at a rate approaching 70 mg/kg, every 24 hours. However, under stressful conditions, or as a result of catecholamine response to shock, these Dextrans are likely to elevate blood glucose levels to potentially harmful limits following intravenous administration, as a response to the rapid degradation of the glucose polymers to free glucose residues.[10–15]

Similar to Dextrans, Hydroxyethyl starches, which are made up of large ethylated starch or glucose polymers, are metabolised by serum amylases to produce smaller molecules of starch polymers and free glucose residues. Even these carry a potential to accelerate blood glucose levels, subsequent to intravenous administration, under stressful conditions.[16–19]

Stress response to surgery and the catecholamine release following it, is itself known to induce some amount of hyperglycaemia, but this remains confined to a limited extent.[20–22] An additive hyperglycemic response, secondary to the metabolism of infused intravenous fluids, can thus prove detrimental to the well-being of the patient, if ignored.

Hyperglycaemia is known to potentiate ischaemic damage to the brain, spinal cord, kidneys, and the myocardium. It is also known to impair wound healing by interfering with the white blood cell function.[22,23] These effects could prove to be even more harmful in fluid resuscitation of uncontrolled diabetics, during neurosurgical procedures, in the event of cardiopulmonary resuscitation or when colloids are included in priming fluids during cardiopulmonary bypass surgeries, using cardioplegia.

Considering these potential ill-effects of hyperglycaemia, in the peri-operative period, on the well-being of patients and on the outcome of surgery, we carried out the following study, with an objective to examine and compare the effects of 6% Hydroxy ethyl starch-450 and Dextran-40, on blood sugar levels, during surgery under spinal anaesthesia, and their potential to induce or potentiate hyperglycaemia.

## METHODS

This prospective, randomised study was carried out, following an approval from the institutional ethics committee. Patients included in this study were informed about the procedure and a written informed consent was taken from all of them.

180, ASA grade 1, non-diabetic patients, 20 to 60 years of age, weighing 40 to 70 kg, undergoing elective lower limb or lower abdominal surgical procedures, which were anticipated to complete within two hours, were selected and randomly divided into three groups, These patients were preloaded with either Ringer’s Lactate (group 1) 20 ml/kg, Dextran-40 (group 2) 10 ml/kg, or 6% Hydroxyethyl starch-450 (group 3) 10 ml/kg, respectively, over a period of 30 minutes, prior to spinal anaesthesia, through an 18-guage intravenous cannula.

Patients on regular use of steroids and ascorbic acid were excluded from the study, as also patients with a low haematocrit (PCV < 30%).All the procedures, which were prolonged due to anaesthetic or surgical complications, or those requiring blood transfusion due to more than predicted intra-operative blood loss were excluded as well.

All the patients were pre-medicated with injection glycopyrrolate 10 μg/kg intravenously before preloading. Basal pulse rate and blood pressure were recorded, and under vigilant monitoring of vital parameters and appearance of skin rash, preloading with the assigned fluid was carried out. Preloading was immediately interrupted on evidence of an allergic reaction and symptomatic treatment was promptly given. Patients developing an allergic reaction to the study fluids were excluded from the study.

Throughout the procedure, capillary blood glucose levels were measured at regular intervals, using a Surestep+ (L-3079RA00903) one-touch glucometer (Lifescan, Johnson and Johnson Laboratories, India). Just prior to the onset of preloading, a baseline reading was noted. This was followed by subsequent readings at 15, 30, 45, 60, 120, 180 and 240 minutes, from the baseline reading. Thus, a total of eight capillary blood glucose readings were recorded.

Following preloading, all the patients received Normal Saline as the subsequent IV maintenance fluid, till the final blood glucose reading was taken. A subarachnoid block was given immediately after completion of the preloading procedure and the level of sensory block after fixation of drug was noted in each case.

### Statistical analysis

For statistical analysis of the clinical data obtained, the Analysis of Variances (ANOVA) with Bonferroni’s post-hoc test was applied, *P* < 0.05 was considered to be significant and *P* < 0.001 was considered to be highly significant.

## RESULTS

The demographic data in all the three groups was comparable, the mean age being 40.7 ± 15.6318 years in group 1, 45.866 ± 14.0901 years in group 2 and 48.0 ± 9.4200 years in group 3. The mean weights in the three groups were 51.333 ± 10.5285 kg, 51.566 ± 3.3803 kg and 51.533 ± 3.7299 kg, respectively [Table 1]. The mean duration of surgery, which was taken as time from surgical incision to skin closure, was also comparable in all the three groups (86.333 ± 23.6691 minutes, 92.666 ± 36.2304 minutes and 96.380 ± 16.3966 minutes, respectively). Mean of the maximum level of sensory blockade achieved after spinal anaesthesia was T6 ± 0.630 in group 1, T7 ± 0.889 in group 2 and T6 ± 10.236 in group 3 [Tables 2 and 3].

**Table 1:** Patient characteristics

Group	Ringer’s lactate (1)	Dextran-40 (2)	Hestar 6%-450 (3)
No. of cases (n)	60	60	60
Mean age (years)	40.7 ± 15.6318	45.866 ± 14.0901	48.0 ± 9420
Male:female	35:25	27:33	17:43
Mean weight (kg)	51.333 ± 10.5285	51.566 ± 3.3803	51.533 ± 3.7299

**Table 2:** Duration of surgical procedure and level of sensory blockade

	Ringer’s lactate (1)	Dextran-40 (2)	Hestar 6%-450 (3)
Mean duration of surgery (min)	86.333 ± 23.66	92.666 ± 36.2304	96.380 ± 16.3966
Mean of maximum level of sensory blockade	T6 ± 0.360	T7 ± 0.889	T6 ± 10.236

**Table 3:** Surgical procedures carried out in the study

Group	Ringer’s lactate (1)	Dextran-40 (2)	Hestar 6%-450 (3)
Orthopaedic	06	10	04
General surgical	30	45	47
Obstetric and gynaecologic	24	05	09

The baseline mean blood glucose levels at onset of preloading in all the three groups were 80.6 ± 16.932 mg/dl (group 1), 78.56 ± 22.661 mg/dl (group 2) and 81.43 ± 16.239 mg/dl (group 3), which were comparable.

**Figure 1:**
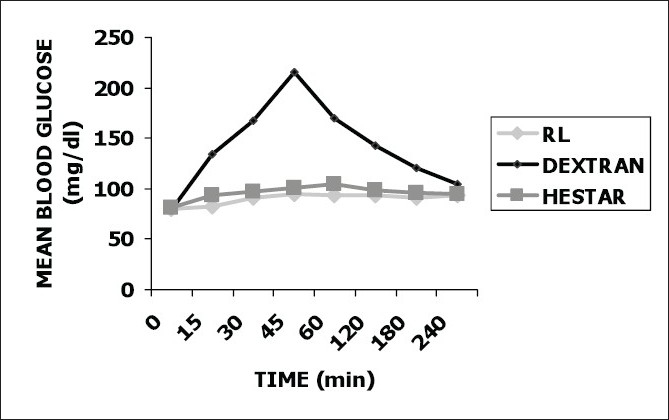
Line diagram showing mean capillary blood glucose levels in all the three groups

In group 1 (Ringer’s Lactate), maximal mean blood glucose levels of 95.16 ± 16.421 mg/dl were found at 45 minutes from onset of preloading, indicating an increase of 14.56 ± 0.511 mg/dl from the baseline, which was found to be a statistically significant rise (*P* < 0.05). After this peak at 45 minutes, the blood sugar levels gradually settled to a mean value of 93.26 ± 12.224 mg/dl at the end of four hours, with a difference of 12.66 ± 4.708 mg/dl from baseline, which was still a statistically significant difference (*P* < 0.05) [Figure 1].

In group 2 (Dextran 40), peak mean capillary blood glucose levels were attained at the end of 45 minutes, a trait similar to that of group 1 (RL). However, the rise in mean capillary blood glucose levels at 45 minutes in the former group was by a huge difference of 36.87 ± 67.820 mg/dl. This leap from the baseline value of 78.56 ± 22.661mg/dl to a mean capillary blood glucose value of 215.43 ± 90.481 mg/dl, at 45 minutes, was found to be statistically highly significant (*P* < 0.001). After this peak at 45 minutes, the mean capillary blood glucose levels gradually fell to a value of 105.5 ± 54.083 mg/dl at the end of four hours. The difference between the former and latter value was of 25.94 ± 31.42 mg/dl, which was still statistically significant (*P* < 0.05).

The trend in group 3 (Hestar450-6%), showed a maximum rise in the mean capillary blood glucose level at the end of the first hour. The difference from the baseline mean capillary blood glucose reading at that time was seen to be 23.83 ± 0.042 mg/dl, which was found to be statistically significant (*P* < 0.05). This was followed by a gradual fall up to 95.42 ± 41.111 mg/dl at the end of four hours.

The difference between the reading at the end of four hours and the baseline reading was of 13.99 ± 2.218 mg/dl. This difference was found to be statistically significant (*P* < 0.05) [Table 4].

**Table 4:** Difference in mean blood glucose levels

Time (minutes)	Ringer’s lactate (1)	Dextran (2)	Hestar (3)
15	1.9 ± 0.944	55.87 ± 20.220**	12.40 ± 1.382*
30	10.36 ± 1.857*	89.26 ± 13.899**	15.287 ± 1.966*
45	14.56 ± 0.511*	136.87 ± 67.820**	19.04 ± 6.014*
60	13.56 ± 0.299*	90.99 ± 60.300**	23.83 ± 0.042*
120	13.16 ± 4.609*	64.80 ± 50.260**	16.79 ± 0.977*
180	10.93 ± 5.690*	42.01 ± 38.490**	14.90 ± 1.957*
240	12.66 ± 4.708*	25.94 ± 31.422*	13.99 ± 2.128*

* P < 0.05

** P < 0.001

## DISCUSSION

A majority of the studies conducted on starch solutions and Dextrans have evaluated their volume expansion properties and their impact on blood coagulation.[24–26] Very few studies have examined the possibility of starches and Dextrans producing hyperglycaemia, in spite of their pharmacodynamic potential to cause the same.

Ringer’s Lactate, used as control in our study, has also been shown to possibly cause hyperglycaemia, due to the conversion of lactate to glucose via the Cori’s cycle.[27]

In the present study, spinal anaesthesia was used as the technique of choice in all the patients, so as to standardise the stress response due to anaesthesia and surgery in all the three groups. For similar reasons, only normal saline was used in all the patients, as the subsequent intravenous fluid, intra-operatively.

Murty *et al*., in 2004, studied the effects of 6% Hestar-450, Pentastarch 200 and Ringer’s Lactate as preloading fluids in spinal anaesthesia, on blood sugar levels. They concluded that both the starches significantly elevated the blood sugar levels (*P* < 0.05), with peaks at the end of two hours with Hestar6%-450 and at the end of three hours with pentastarch 6%-200. However, in their study, Ringer’s Lactate did not significantly elevate blood sugar levels.[17]

Our study demonstrated a sustained and statistically significant rise (*P* < 0.05) in blood sugar levels from the baseline, with the infusion of both Ringer’s lactate and Hydroxy ethyl starch 6%-450, which peaked at the end of 45minutes and at the end of one hour, respectively. Dextran 40, on the other hand demonstrated a steep and statistically highly significant rise (*P* < 0.001) in mean capillary blood glucose levels from the mean baseline reading, which peaked at 45 minutes.

In the first 15 minutes of infusion, when there was no stress owing to anaesthesia or surgery, there were different trends seen in the three study groups. In group 1(R L), the rise in mean capillary blood glucose level was only 1.9 ± 0.944 mg/dl, which was statistically not significant, whereas, in group 2 (Dextran), increase in mean blood glucose levels in the first 15 minutes was 55.87 ± 20.220 mg/dl, which was statistically highly significant (*P* < 0.001). In group 3 (Hestar) the difference in mean blood glucose levels at 15 minutes from baseline was 12.40 ± 1.382 mg/dl (*P* < 0.05).

When the clinical data obtained in group 3 (Hestar) was compared with that of group 1 (RL), it was found to be comparable throughout the period of infusion, excepting the early rise in blood glucose level seen with the Hestar group.

When the trends in group 2 (Dextran) were compared with those seen in group 3 (Hestar), a statistically highly significant rise was seen in the difference in mean blood glucose readings from the baseline values, at various time intervals, in group 2 as compared to group 3 (*P* < 0.001). The mean blood glucose reading at the end of four hours in group 3 was 95.42 ± 14.111 mg/dl, while in group 2 it was 104.5 ± 54.083 mg/dl; the difference between the two being statistically significant (*P* < 0.05).

The mean readings at the end of four hours in all the three study groups were significantly higher than their respective mean baseline blood glucose readings in all the three groups.

## CONCLUSION

We thus conclude that, under stressful conditions, Ringer’s lactate and Hydroxyethyl starch 6%-450 significantly raise the blood sugar level, albeit within physiological limits, whereas, Dextran-40 raises the blood sugar to a level which is well above the physiological limit.

Our study has a few limitations, though; we included hydroxyethyl starch 6%-450 and Dextran-40, as study infusions, primarily because of their relatively easier availability in our institution, leaving scope for inclusion of other starches and mucopolysaccharides with different molecular weights and different compositions in future studies. Similarly, we included only non-diabetic adult patients in our study, and thus, would like to recommend inclusion of diabetic patients for future research. There is scope for further research on this subject in areas where hyperglycaemia is preferably avoided, but colloids are regularly used, such as, in neurosurgical procedures, open heart surgeries, cardiopulmonary resuscitation, and so on.
